# Political Landmines in Healthcare Research: An Obstacle to Progress

**DOI:** 10.7759/cureus.66707

**Published:** 2024-08-12

**Authors:** Abdulqadir J Nashwan

**Affiliations:** 1 Nursing & Midwifery Research Department, Hamad Medical Corporation, Doha, QAT

**Keywords:** international collaboration, regulatory barriers, research funding, healthcare research, political landmines

## Abstract

The quest for groundbreaking discoveries in healthcare research faces significant challenges, not just technical but also political. Political landmines, such as controversies, regulations, and policies influenced by political agendas, affect healthcare research. These landmines can derail studies, stifle innovation, and impede the advancement of medical knowledge and public health. Political agendas often impose narratives that contradict scientific evidence, influencing research areas like reproductive health, climate change, and vaccinations. Funding volatility due to political shifts creates uncertainty, discouraging long-term projects and slowing healthcare innovation. Ethical and regulatory barriers shaped by political considerations further limit research scope and delay breakthroughs. Political influences also result in censorship and misinformation, undermining informed decision-making and public trust. Geopolitical tensions hinder international collaboration, reducing the capacity to address global health challenges. To mitigate these effects, the scientific community must advocate for evidence-based policies, communicate transparently with policymakers, and build robust alliances to support research independence. Fostering resilience within the research community is crucial for adapting to changing political climates and ensuring the continuity of essential projects. Healthcare research can continue to advance and improve global health outcomes by addressing these political challenges.

## Editorial

The quest for groundbreaking discoveries and innovations is often fraught with challenges in healthcare research [1]. While technical and scientific hurdles are expected and addressed through rigorous methodologies and persistent inquiry, an insidious and often underestimated obstacle lies in the political landscape. Political landmines - controversies, regulations, and policies influenced by political agendas - jeopardize healthcare research's integrity, progress, and impact [2]. These landmines can derail studies, stifle innovation, and ultimately impede the advancement of medical knowledge and public health.

One of the most pervasive ways political landmines impact healthcare research is by imposing agendas that do not align with scientific evidence or public health needs. Political figures and parties may have vested interests in promoting certain narratives or policies that serve their goals, even if they contradict empirical data [3]. For instance, research into areas like reproductive health, abortion, climate change, and vaccinations has often been hindered by political agendas that either deny established science or push for the suppression of findings that may be deemed controversial. This intrusion compromises the objectivity of research, leading to biased outcomes that fail to serve the best interests of public health.

Healthcare research relies heavily on funding, much of which comes from government sources. Political instability or shifts in power can lead to abrupt changes in funding priorities [4]. A newly elected administration may cut funding for specific research areas to favor others that align with their political ideologies. This volatility creates an environment of uncertainty, discouraging researchers from pursuing long-term projects in regions that might become politically unfavorable. Consistency in funding disrupts ongoing studies and discourages new research initiatives, slowing the overall progress of healthcare innovation.

Political influences often manifest as ethical and regulatory barriers that complicate or prohibit certain research types. Stem cell research, for example, has long been a contentious issue in many countries, with political and religious beliefs shaping the legal framework governing it. These restrictions can severely limit the scope of research and delay potential medical breakthroughs. Similarly, regulatory frameworks influenced by political considerations can impose excessive bureaucracy and stringent requirements that stifle innovative approaches and experimental treatments [5]. The result is a research environment where scientific inquiry is subordinated to political expediency.

In some cases, political landmines result in the censorship of research findings or the dissemination of misinformation. Governments or influential political entities may suppress studies that challenge their policies or contradict their public messaging. This censorship prevents the public and the scientific community from accessing vital information, undermining informed decision-making and public trust in science. Additionally, the spread of misinformation, often fueled by political motives, can distort public perception of scientific issues, leading to resistance against evidence-based interventions such as vaccinations or public health measures during pandemics.

Healthcare research increasingly relies on international collaboration, pooling resources, expertise, and data from across the globe. However, geopolitical tensions and protectionist policies can hinder these collaborations (e.g., COVID-19). Restrictions on data sharing, travel bans, and political conflicts can prevent researchers from different countries from working together effectively. The fragmentation of international research efforts reduces the collective capacity to address global health challenges, such as pandemics, antimicrobial resistance, and chronic diseases, which require coordinated and comprehensive approaches.

To mitigate the detrimental effects of political landmines, the scientific community must advocate for policies prioritizing evidence-based research and public health above political considerations. Researchers should communicate transparently with policymakers and the public, emphasizing scientific integrity's importance and unbiased research's long-term benefits. Building robust alliances with international research bodies, non-governmental organizations, and private sector partners can also provide alternative funding sources and support systems, reducing reliance on politically driven government funding.

Furthermore, fostering a culture of resilience within the research community is crucial. Researchers must be prepared to adapt to changing political climates and advocate for the continuity and stability of essential research projects. The healthcare research community can continue pursuing advancements that enhance global health outcomes despite the ever-present threat of political obstacles by staying watchful and taking proactive steps to address political challenges.

In conclusion, political landmines represent a significant and multifaceted challenge to healthcare research. Their impact ranges from funding instability and regulatory barriers to censorship and international collaboration obstacles. Addressing these challenges requires a concerted effort from the scientific community, policymakers, and the public to ensure that healthcare research remains driven by evidence, free from undue political influence, and focused on advancing human health and well-being.
